# The Hidden Link Between Intestinal Helminthiasis, Gut Microbiome Alterations, and Colorectal Cancer Risk: A Systematic Review

**DOI:** 10.3390/ijms27114957

**Published:** 2026-05-29

**Authors:** Dieketseng Palesa Shemfe, Nontobeko Eunice Mvubu, Pragalathan Naidoo, Jennifer Giandhari, Doratha Armen Byrd, Sayed Shakeel Kader, Zilungile Lynette Mkhize-Kwitshana

**Affiliations:** 1 Department of Medical Microbiology, School of Medicine, College of Health Sciences, Nelson R. Mandela School of Medicine, University of KwaZulu-Natal, Durban 4001, South Africa; naidoop5@ukzn.ac.za (P.N.); mkhizekwitshanaz@ukzn.ac.za (Z.L.M.-K.); 2 Division of Research Capacity Development, South African Medical Research Council (SAMRC), Tygerberg, Cape Town 7505, South Africa; 3 Department of Medical Biosciences, Faculty of Natural Sciences, University of the Western Cape, Cape Town 7535, South Africa; 4 KwaZulu-Natal Research Innovation and Sequencing Platform, School of Medicine, College of Health Sciences, Nelson R. Mandela School of Medicine, University of KwaZulu-Natal, Durban 4001, South Africa; giandharij@ukzn.ac.za; 5 Department of Cancer Epidemiology, H. Lee Moffitt Cancer Center and Research Institute, Tampa, FL 33606, USA; doratha.byrd@moffitt.org; 6 Department of Surgery, University of KwaZulu-Natal, Durban 4001, South Africa; shakeelkader2006@gmail.com; 7 Biomedical Sciences Department, School of Life and Consumer Sciences, College of Agriculture and Environmental Sciences, University of South Africa, Florida Campus, Johannesburg 1710, South Africa

**Keywords:** neglected tropical disease, soil-transmitted helminths, helminthiasis, gut microbiota, dysbiosis, colorectal cancer, microbial diversity, inflammation

## Abstract

Colorectal cancer (CRC) is an increasing health concern in low- and middle-income countries (LMICs), especially in Africa, driven by dietary shifts, urbanisation, infections, and limited treatment access. The gut microbiome plays a central role in CRC, while soil-transmitted helminths (STHs) exert complex effects that can promote or mitigate risk depending on species, infection intensity, and host context. This systematic review synthesised 17 human studies (2000–2026) examining helminth impacts on gut microbial diversity, revealing a dualistic pattern. Several studies reported that chronic or moderate helminth infections, such as *Ascaris lumbricoides* and *Trichuris trichiura*, were associated with increased bacterial richness and the expansion of beneficial taxa, including Paraprevotellaceae, *Parabacteroides*, *Agathobacter*, Ruminococcaceae, and *Lactobacillus*. These taxa are associated with the production of short-chain fatty acids (SCFAs), protection of the epithelial barrier, and regulation of the immune system, suggesting a potential buffering effect against inflammation-driven carcinogenesis. On the contrary, other studies demonstrated helminth-associated dysbiosis characterised by reduced diversity and enrichment of pro-inflammatory and oncogenic taxa. *T*. *trichiura* and *Strongyloides stercoralis* infections were associated with the expansion of *Treponema succinifaciens*, *Streptococcus gallolyticus*, Enterobacteriaceae, and *Ruminococcus torques*, which are linked to reduced gut microbiome diversity, pro-inflammatory states, and oncogenic processes. Furthermore, *A*. *lumbricoides* infections altered the host microbiome at the phylum level, with increased Proteobacteria and reduced Firmicutes and Bacteroidetes, alongside metabolome shifts in amino acid and lipid pathways that have been associated with tumourigenic processes. Collectively, the evidence shows that helminthiasis may either enrich potentially protective microbes or be associated with pro-tumourigenic dysbiosis, with outcomes shaped by species, infection intensity, and host context. Notably, none of the included studies directly assessed CRC, underscoring the fact that current evidence is indirect and mechanistic. Overall, helminths are associated with gut microbiome shifts in both potentially protective and potentially harmful directions. This systematic synthesis of human evidence provides an integrated understanding of how helminth-associated microbiome shifts may influence colorectal carcinogenesis and highlights the need for longitudinal mechanistic studies to clarify causality and inform biomarker discovery and prevention in endemic regions.

## 1. Introduction

Helminthiasis remains one of the most prevalent parasitic diseases globally, with an estimated 1.5 billion people worldwide infected with soil-transmitted helminths (STHs), primarily in tropical and subtropical regions [1]. The highest prevalence is reported in Sub-Saharan Africa, East Asia, and parts of South America, where inadequate sanitation, unsafe water, and overcrowding facilitate transmission [2]. Epidemiological studies have directly linked helminth infection rates to poor hygiene infrastructure; for instance, Pullan and Smith [3] found prevalence strongly correlating with inadequate sanitation and population density, while Freeman and Chard [4] showed significantly higher STH infection rates among Kenyan children lacking access to clean water. Beyond their transmission dynamics and health impacts, helminth infections are increasingly recognised for their complex interactions with host biology, particularly their ability to reshape the gut microbiome [5].

These alterations occur through direct mechanisms such as increased mucus secretion, antimicrobial peptide release, altered gut motility, and enhanced epithelial permeability [6,7,8,9]. Additionally, these infections release excretory-secretory products (ESPs), including bioactive antimicrobial molecules that selectively inhibit or promote specific bacterial taxa [10]. Indirectly, helminths provoke potent host immune modulation, typically skewing host responses toward T-helper type 2 (Th2) immunity and regulatory T-cell (Treg)-associated immune responses that influence microbial community structure and colorectal cancer (CRC)-relevant immune pathways [11,12]. In CRC, Treg accumulation can suppress anti-tumour immune surveillance by weakening effector T-cell function, particularly clusters of differentiation 8-positive (CD8^+^) T-cell-mediated anti-tumour activity, thereby enabling transformed or malignant epithelial cells to evade immune clearance and supporting tumour immune escape and progression [13,14]. This mechanism is supported by CRC-specific evidence showing that targeting tumour-infiltrating Tregs restores CD8^+^ T-cell function and induces anti-tumour immunity [15].

In parallel, persistent Th2-type signalling may favour alternatively activated, M2-like macrophage responses. Although these responses are normally involved in tissue repair and immune regulation, within the colorectal tumour microenvironment, they can become tumour-supportive by promoting immunosuppression, angiogenesis, tissue remodelling, invasion and metastatic potential [16,17,18]. Thus, helminth-induced Th2/Treg polarisation provides a plausible, context-dependent mechanistic link between infection-driven immune regulation, dysbiosis and CRC susceptibility by potentially weakening anti-tumour immune surveillance while favouring immune-regulatory, tissue-remodelling and stromal conditions that may become tumour-supportive within the colorectal tumour microenvironment. These shifts have been observed in helminth-endemic populations; for instance, Zaiss, Rapin [19] reported enrichment of short-chain fatty acid (SCFA)-producing bacteria that support immune regulation, while Ramanan, Bowcutt [20] observed the expansion of Clostridiales and depletion of Bacteroidales that reversed after deworming. Collectively, such helminth-driven microbiome changes may create a permissive intestinal niche for parasite survival, but they also raise concerns for long-term host health, including CRC.

CRC is the third most commonly diagnosed cancer and the second leading cause of cancer deaths worldwide, with approximately 1.93 million new cases and 0.94 million deaths in 2020 [21,22]. Its burden continues to rise by 2–4% annually in many low- and middle-income countries (LMICs), including regions where helminths remain endemic [23]. This trend reflects broader epidemiological transitions, driven by dietary shifts, urbanisation, and changing microbial exposures. The gut microbiome has emerged as a central factor in CRC pathogenesis, influencing disease development through the production of genotoxins, modulation of inflammation, and disruption of epithelial barrier integrity. However, gut microbial composition is not shaped by disease alone; dietary patterns, antibiotic exposure, geographic settings, and host genetic backgrounds are well-established independent determinants of microbiome structure that must be considered when interpreting helminth-associated microbial changes [24,25].

In recent years, numerous studies have shown that the gut microbiome of CRC patients differs markedly from that of healthy individuals [26,27,28]. Extensive case–control studies have further demonstrated that both young and old-onset CRC patients harbour similarly altered gut microbiome profiles, distinctly different from healthy controls and characterised by enrichment of known pathogenic taxa [29,30]. These tumour-associated microbial shifts collectively promote inflammation, genotoxicity, and immune evasion [31,32,33]. Large-scale metagenomic studies have confirmed these patterns. In a pooled analysis of 3741 stool metagenomes from 18 case–control studies, Piccinno and Thompson [34] demonstrated that gut microbiome profiles could discriminate CRC patients from healthy controls with high accuracy (area under the ROC curve = 0.85). Piccinno and Thompson [34] further showed that enrichment of *Fusobacterium nucleatum* is a consistent CRC feature, contributing substantially to the predictive microbiome signature. Similarly, the Zepeda-Rivera and Minot [35] study, comparing 627 CRC patients and 619 healthy controls, identified a clade of *F*. *nucleatum* (subspecies *animalis* clade C2) that was significantly overrepresented in both tumour tissues and stool samples.

Beyond *F*. *nucleatum*, multiple other pathogens have been implicated in CRC. Enterotoxigenic *Bacteroides fragilis* (ETBF) secretes *B. fragilis* toxin (BFT), inducing DNA damage and colonic inflammation [36]. Adherent-invasive *Escherichia coli* carrying the pks island produce colibactin, a genotoxin that causes DNA double-strand breaks [37]. *Enterococcus faecalis* generates extracellular reactive oxygen species that impair DNA repair and promote genomic instability [38]. *Peptostreptococcus* has likewise been associated with CRC, as it is enriched in tumours and stools and linked to pro-inflammatory dysbiosis [39]. Collectively, these pathogens reduce protective commensals, fostering a pro-carcinogenic microbiome. Consistent with this, large-scale studies have reported reduced bacterial α-diversity (a measure of species richness and evenness within individual samples) in CRC patients alongside enrichment of pro-inflammatory taxa [40,41,42]. From a prognostic perspective [39], this demonstrates that higher faecal microbial α-diversity is associated with significantly improved disease-free survival among stage I–III CRC patients (hazard ratio [HR] = 0.40; 95% confidence interval [CI] 0.19–0.87; *p* = 0.02), while at the taxa level, *Peptostreptococcus* predicts worse outcomes (HR = 1.62; *p* = 0.01) and Clostridiales are associated with better survival (HR = 0.62; *p* = 0.01). Despite these advances, the role of helminths in shaping CRC-relevant microbiome alterations remains poorly understood.

Human studies provide early evidence of this link. Hookworm-infected adults in Ghana exhibited higher bacterial richness and restructuring of gut communities compared to uninfected peers [43]. Ethiopian children harbouring *Trichuris trichiura* displayed lower α-diversity and distinct compositional shifts [44]. In Uganda, *Schistosoma mansoni* infection was associated with elevated faecal calprotectin and occult blood, which are markers of intestinal inflammation [45]. Similarly, Amaruddin and Koopman [46] reported increased intestinal permeability among Indonesian children with STHs, as indicated by higher lactulose:mannitol ratios and elevated plasma intestinal fatty-acid binding protein (I-FABP) levels, which serve as a marker for intestinal injury or inflammation. Yet, the direct mechanistic contribution of helminth-driven dysbiosis to CRC remains insufficiently characterised. Reference [47] proposed immune pathways linking helminth infection to CRC progression, while [48] described overlaps between parasitic infection and microbiota profiles in Malaysian CRC patients. However, neither study directly investigated microbiome alterations as a causal mechanism of CRC.

This review addresses this critical gap through two related but distinct aims: first, the aim is to systematically synthesise human evidence on helminth-associated alterations in gut microbiome composition, diversity, and functional capacity across endemic and non-endemic populations; the second aim is to evaluate the biological plausibility of mechanistic pathways through which such microbiome shifts may influence colorectal carcinogenesis. Given that none of the included studies directly assessed CRC endpoints, the second aim is necessarily inferential, drawing on established microbiome–cancer mechanisms to contextualise the helminth-associated microbial patterns identified in the included studies. Clarifying this helminth–microbiome–cancer relationship is essential for identifying biomarkers and informing prevention strategies, particularly in helminth-endemic LMICs where chronic infections coincide with a rising CRC burden.

## 2. Methods

### 2.1. Literature Search Strategy

A systematic review of the current literature examining the association between alterations in the gut microbiome during intestinal helminth infections and the potential risk of colorectal cancer was conducted. Searches were performed in the databases ScienceDirect, Google Scholar, MEDLINE, PubMed, and the Institute for Scientific Information (ISI) Web of Knowledge.

A comprehensive search strategy was developed using Boolean operators combining three domains: (i) helminth-related terms, (ii) gut microbiome-related terms, and (iii) colorectal cancer-related terms. The sample search string used in PubMed was: (“helminth infection” OR “soil-transmitted helminths” OR “intestinal parasites”) AND (“gut microbiome” OR “gut microbiota” OR “bacterial diversity”) AND (“colorectal cancer” OR “colon cancer” OR “tumorigenesis”). The search strategy was adapted across databases.

**Figure 1:**
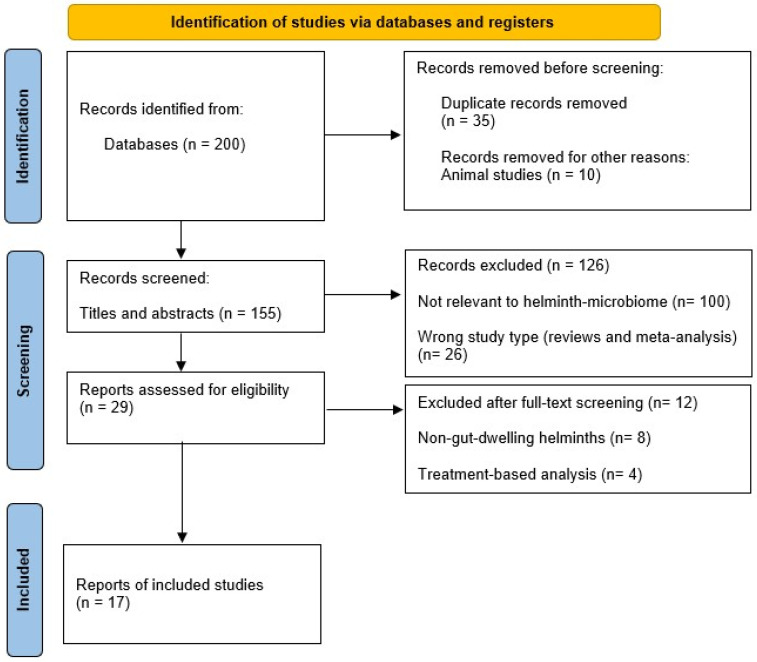
PRISMA 2020 flow diagram showing the study selection process for the systematic review titled “The Impact of Helminth Infections on the Gut Microbiome and Its Potential Implications for Colorectal Cancer: A Systematic Review.” A total of 29 full-text articles were assessed for eligibility after initial screening from databases including PubMed, ScienceDirect, Google Scholar, MEDLINE, and ISI Web of Knowledge. Following the inclusion and exclusion criteria, 17 original research articles were included in the final synthesis. Reasons for exclusion at each stage are detailed in the diagram.

Keywords used included terms related to the gut microbiome (“gut microbiota,” “bacterial diversity,” “microbial dysbiosis”), helminth infections (“helminth infection,” “soil-transmitted helminths,” “intestinal parasites,” “worms”), and colorectal cancer (“colorectal cancer,” “colon cancer,” “tumorigenesis,” “intestinal carcinogenesis”). To maximise sensitivity, these were expanded to include additional helminth-specific terms (e.g., hookworm, *Ascariasis*, *Trichuriasis*, *Schistosomiasis*, Strongyloidiasis), microbiome-related terms (e.g., “microbial community,” “16S rRNA sequencing,” metagenomics, qPCR), and cancer-related synonyms (e.g., “colorectal neoplasm,” “bowel cancer,” CRC). Relevant articles identified through this comprehensive search were evaluated and documented following PRISMA guidelines [49], as illustrated in Figure 1.

### 2.2. Study Selection, Quality of Studies, and Data Extraction

The primary author (D.P.S.) initially screened the literature by reviewing titles, abstracts, and full texts according to predefined inclusion and exclusion criteria. The six co-authors (N.E.M., P.N., J.G., D.A.B., S.S.K. and Z.L.M.K.) conducted an additional assessment of the selected studies’ eligibility for inclusion in this systematic review, identifying discrepancies and eliminating duplicates. The extracted data quality was classified into four tiers: high, moderate, poor, and very low-quality (Group, 2004). This classification was evaluated utilising the GRADE (Grading of Recommendations, Assessment, Development, and Evaluations) methodology [50]. The quality assessments informed the interpretation of findings presented in the Results and Discussion sections.

Inclusion criteria:

- Studies in the literature addressing helminth infections, the diversity of the gut microbiome, and CRC:

Peer-reviewed publications investigating the correlation between helminth infections and gut microbiome diversity in human subjects. Investigations assessing the impact of intestinal helminth species on gut microbiome, including alterations in microbial composition and diversity, and the potential elevation in CRC risk.

- Language: Only articles published in English were considered.
- Population: Research encompassing human participants of all age demographics.
- Study Design: Observational studies, clinical trials, cohort studies, cross-sectional studies, and case–control studies.
- Publication Period: January 2000 to March 2026.

Exclusion criteria:

- Publication Date: Studies in the literature published before January 2000.
- Language: Literature not published in English.
- Study Design: Systematic reviews, meta-analyses, editorials, conference abstracts, and animal studies.
- Relevance: Studies that do not specifically address the association between helminth infection, gut microbiome diversity, and CRC risk.

## 3. Results

### 3.1. Study Selection

A total of seventeen original research articles met the predefined inclusion criteria and were included in this systematic review, following PRISMA-guided screening and eligibility assessment.

### 3.2. Study Characteristics

The included studies (*n* = 17) represented a broad geographical distribution, encompassing populations from Southeast Asia (Thailand, Malaysia, and Indonesia), sub-Saharan Africa (Tanzania and Ethiopia), South America (Ecuador), and non-endemic Western settings (Netherlands, United Kingdom, and Italy). Sample sizes varied substantially across studies, ranging from 10 to 650 participants, reflecting heterogeneity in study scale and design. Across the included studies, the majority focused on soil-transmitted helminths, particularly *Ascaris lumbricoides*, *T*. *trichiura*, and *Necator americanus*. Mixed helminth infections were reported in 10 of the 17 studies, whereas fewer studies investigated single-species infections, including *Strongyloides stercoralis* (*n* = 3) and *T*. *trichiura* (*n* = 1), highlighting variability in infection profiles across populations.

Regarding study design, cross-sectional studies were the most common (*n* = 9), followed by longitudinal designs (*n* = 4), randomised controlled trials (*n* = 3), and experimental infection studies (*n* = 2). This distribution indicates a predominance of observational evidence, with relatively limited longitudinal and experimental data to support causal inference. Experimental studies involving controlled *N*. *americanus* infection provided insight into microbiome responses under defined exposure conditions. In terms of microbiome profiling, 16S rRNA gene sequencing was employed in the majority of studies (*n* = 14), while shotgun metagenomics was used in two studies, and one study utilised both 16S rRNA sequencing and shotgun metagenomics. Across all included studies (*n* = 17), stool samples constituted the primary biological material analysed. However, a subset of studies incorporated additional sample types, including helminth gut material (*n* = 2) and saliva (*n* = 1), to complement microbial analyses.

Overall, the methodological quality of included studies ranged from moderate to high, although many were limited by small sample sizes and cross-sectional designs, which constrained causal interpretation. A detailed summary of study characteristics is presented in Table 1, while gut microbiome outcomes, including diversity, taxonomic composition, and clinical associations, are summarised in Table 2.

## 4. Discussion

This systematic review evaluated how helminth infections influence the gut microbiome and explored their potential implications for CRC. Across 17 studies, the evidence demonstrates that helminths profoundly alter microbial richness, composition, and metabolic function. However, these effects are highly variable, with some patterns suggesting protective roles against carcinogenesis, including enrichment of SCFA-producing taxa and microbial diversity [51,53,55,61,65] and others pointing to pro-inflammatory and pro-tumourigenic consequences, such as expansion of *S*. *gallalyticus*, Enterobacteriaceae, and mucin-degrading species [44,59,60,61]. Overall, the evidence base is characterised by heterogeneity in study design, population, and analytical approaches, which should be considered when interpreting these findings.

### 4.1. Protective Impacts of Helminths on the Microbiome and CRC Risk

Several of the included studies reported that helminth infections enhance bacterial diversity and enrich taxa with potential protective roles. *T*. *trichiura* colonisation among rural Malaysians was associated with increased microbial richness and enrichment of pathways related to genetic information processing, specifically DNA repair, replication, and transcriptional regulation [55]. These functions are essential for maintaining microbial genome stability and metabolic adaptability, which may support ecological resilience within the gut. Such resilience is thought to buffer against dysbiosis and chronic inflammation, both of which are key hallmarks implicated in colorectal carcinogenesis [66,67]. In a controlled trial, *N*. *americanus* infection was associated with bacterial expansion during the chronic phase of infection, suggesting the establishment of a stable host–microbiota equilibrium [51]. Similarly, multiple sclerosis patients with hookworm maintained stable microbial diversity with expansion of *Parabacteroides* and Tenericutes, which are taxa linked to immune modulation through enhanced Treg activity and suppression of pro-inflammatory cytokines such as IL-6 and TNF-α [53]. These immunomodulatory effects may be associated with reduced chronic intestinal inflammation and restoration of mucosal immune tolerance, potentially lowering the risk of inflammation-driven colorectal carcinogenesis. Importantly, these findings suggest that helminth-induced increases in microbial diversity and functional capacity contribute to ecological stability within the gut, a factor consistently associated with resistance to dysbiosis and inflammation-driven carcinogenesis.

For instance, *Agathobacter*, a known butyrate producer, was enriched in Ethiopian children with *T. trichiura* [44]. Because butyrate supports epithelial integrity and exerts anti-inflammatory effects, its presence is generally interpreted as beneficial [68,69,70]. However, whether this enrichment reflects a protective response or simply a feature of helminth-associated dysbiosis remains unclear. At the same time, the study reported reduced diversity and enrichment of other taxa with pro-carcinogenic associations (see Section 4.2). Collectively, these findings indicate that helminth-driven microbiome shifts are not uniformly beneficial or harmful, but instead reflect a complex, context-dependent reorganisation of the microbial community. Helminth-positive Malaysian villages exhibited Firmicutes-rich microbiomes, which is a finding of potential significance because many Firmicutes genera (such as *Faecalibacterium* and *Roseburia*) are established butyrate producers, and their enrichment has been associated with SCFA-mediated protection against CRC [71,72]. Studies on *S*. *stercoralis* demonstrated an increase in Paraprevotellaceae and Ruminococcaceae [61]. While Ruminococcaceae are well-recognised SCFA producers linked to barrier reinforcement and immune regulation [70,71,72], the role of Paraprevotellaceae in CRC protection is less clear, highlighting the heterogeneity of helminth-induced microbial shifts.

Complementing these findings, Nguyen and Hongsrichan [73] reported that individuals with *S*. *stercoralis* from Thailand exhibited decreased serum acetic acid despite increased microbial richness, suggesting that helminth infection may alter SCFA metabolism in a context-dependent manner. Together, these findings indicate that helminth colonisation can foster bacterial communities capable of producing SCFAs such as butyrate and propionate. These metabolites play critical anti-carcinogenic roles by supporting epithelial integrity, suppressing pro-inflammatory cytokines, and inducing apoptosis in colonocytes, functions that have been well-characterised in human studies [70]. Collectively, these data suggest that in certain contexts, helminths may buffer against dysbiosis and reduce CRC initiation risk by reinforcing microbial stability and anti-inflammatory function.

Most studies included in this systematic review reported that helminthiasis was also associated with an enrichment of SCFA-related lineages, indicating not only an enhanced microbial fermentation capacity but also potential protective effects against colorectal carcinogenesis. SCFAs, particularly butyrate and propionate, are bioactive metabolites that regulate gut homeostasis through multiple tumour-suppressive mechanisms. First, they reinforce the intestinal epithelial barrier by upregulating tight junction proteins such as claudin-1 (a transmembrane protein regulating paracellular permeability, often dysregulated in CRC) and zonula occludens-1 (ZO-1), a scaffolding protein that stabilises tight junctions, the loss of expression of which is associated with barrier dysfunction and CRC progression. In support of this, Yan and Ajuwon [74] demonstrated that exposure to butyrate in intestinal epithelial cells significantly increased the expression of claudin-1 and ZO-1, while enhancing transepithelial electrical resistance (TEER) as a functional indicator of barrier integrity.

In addition, SCFAs enhance mucin 2 (MUC2) production (a major component of the intestinal mucus layer, the reduction of which compromises barrier function and predisposes to tumourigenesis) [70,75]. Secondly, SCFAs exert potent anti-inflammatory effects by inhibiting nuclear factor kappa-light-chain-enhancers of activated B cells (NF-κB) (a transcription factor central to inflammatory signalling) and reducing pro-inflammatory cytokines such as interleukin-6 (IL-6) and tumour necrosis factor-alpha (TNF-α), while simultaneously promoting the expansion of Tregs(a T-cell subset that produces IL-10 and maintains immune tolerance) [24,71]. Thirdly, butyrate induces apoptosis in transformed colonocytes by acting as a histone deacetylase (HDAC) inhibitor (enzymes with inhibition that lead to chromatin relaxation, cell cycle arrest, and programmed cell death of cancer cells) [24]. Finally, SCFAs modulate key oncogenic pathways, including Wnt/β-catenin (a signalling cascade that regulates intestinal stem cell renewal and proliferation, and is frequently hyperactivated in CRC) and phosphoinositide 3-kinase/protein kinase B (PI3K/Akt) (a survival pathway regulating growth and apoptosis resistance, often upregulated in CRC), thereby restraining tumour-promoting signals [74,76]. Collectively, these findings suggest that helminth-driven enrichment of SCFA-producing taxa could counterbalance oncogenic pressures by strengthening epithelial integrity, dampening inflammation, and activating tumour-suppressive signalling cascades. However, despite these well-established anti-carcinogenic mechanisms, direct evidence linking helminth-associated enrichment of SCFA-producing taxa to reduced CRC risk in humans remains limited, as none of the included studies directly assessed cancer outcomes. Furthermore, the assumption that enrichment of SCFA-producing taxa is uniformly protective warrants nuance. Microbial functional redundancy means that taxonomic presence does not reliably predict metabolic output, and 16S rRNA amplicon sequencing, the predominant method used across the included studies, cannot confirm in situ SCFA production or distinguish functionally active from dormant populations. Helminth-associated enrichment of putative SCFA producers should therefore be interpreted as indicative of potential rather than confirmed anti-carcinogenic activity.

### 4.2. Harmful and Pro-Carcinogenic Impacts of Helminths

In contrast, several included studies demonstrated that helminth infections may foster microbial profiles associated with inflammation and carcinogenesis. In Ethiopian schoolchildren, *T. trichiura* infection was linked to reduced microbial diversity, marked by loss of beneficial *Prevotella* spp. and enrichment of taxa such as *Agathobacter*, *T*. *succinifaciens*, and *Streptococcus gallolyticus* [44]. While *Agathobacter* is typically described in the literature as a butyrate-producing genus associated with gut health, its enrichment here occurred in a setting of reduced diversity and co-expansion of pro-carcinogenic taxa such as *S*. *gallolyticus*. This suggests that its function in helminth-associated dysbiosis may be context-dependent and should not be assumed to confer protection in this scenario.

Similarly, Tanzanian women with *T. trichiura* showed depletion of probiotic genera (*Weissella* and *Leuconostoc*) and enrichment of pathogenic pathways, including increased *S. gallolyticus*, a species strongly associated with human colorectal neoplasia [59]. Clinical evidence demonstrates that *S*. *gallolyticus bacteremia* and endocarditis are strongly associated with undiagnosed CRC or advanced adenomas, as suggested by Boleij and van Gelder [77], while functional studies suggest that *S*. *gallolyticus* can directly promote colon cancer cell proliferation and tumour growth via β-catenin activation, as suggested by Kumar and Herold [78], underlining its carcinogenic relevance. Rosa and Supali [60] reported the enrichment of *Enterococcus*, *Flavonifractor*, and *Olsenella* in individuals heavily infected in Liberia and Indonesia; these taxa were associated with genotoxic metabolite production and chronic mucosal inflammation. Tran and Chaidee [61] further showed that chronic *S. stercoralis* infection selectively enriched *Ruminococcus torques*, a mucin-degrading species linked to barrier erosion and CRC, while reducing carbohydrate metabolism post-treatment, reflecting functional instability. *A. lumbricoides* infections also carry risk. Muslim and Aazmi [58] observed altered host microbial profiles in *A. lumbricoides*-infected individuals, while Klomkliew and Sawaswong [54] found that severe *Ascaris* cases were characterised by elevated amino acids, lipids, and nucleotide precursors in parasite metabolomes, potentially creating nutrient-rich, pro-growth environments in the gut.

Epidemiological evidence supports a connection between chronic schistosomiasis and CRC risk. Reviews of human cases with *Schistosoma japonicum* highlight frequent co-occurrence with CRC, as reported by Salim and Hamid [79], and a retrospective analysis found schistosomiasis to be an independent predictor of poor overall survival in CRC patients, especially in advanced stages [80]. Additionally, systematic human-focused syntheses highlight the broader impact of schistosomiasis-induced inflammation, which has been associated with microbial and carcinogenic shifts in the colon [81]. Collectively, these findings suggest that helminth-induced dysbiosis, particularly under conditions of high burden or chronicity, may be associated with oncogenic processes through sustained inflammation, epithelial damage, metabolic reprogramming, and impaired immune surveillance.

### 4.3. Context Dependence of Helminth–Microbiome Interactions

The divergent findings across studies emphasise that the effects of helminths on the microbiome and CRC risk are highly context-dependent. Beyond immune polarisation, ESPs may directly modify mucosal conditions, including epithelial permeability, mucus glycoprotein composition, and antimicrobial peptide output, that govern selective pressure on luminal microbial communities, providing a helminth-specific mechanistic basis for the compositional shifts documented across the included studies. Martin and Kaisar [57] reported that helminth infection altered the normal relationship between microbial composition and cytokine responses. In participants negative for helminth, higher bacterial diversity correlated with stronger IFN-γ responses to phytohaemagglutinin (PHA) stimulation, while higher proportions of Bacteroidetes were associated with lower IL-10 responses to LPS stimulation; both relationships were weakened in helminth-positive individuals. Lee and Tang [25] demonstrated that helminth infection exerted greater effects on microbial composition than either diet or blood biochemistry in indigenous Malaysian individuals. The infections were associated with changes in systemic zinc and iron, both of which are critical for immune and redox balance, and with taxonomic shifts involving Clostridiales and Bacteroidales.

Notably, not all helminths produced disruption of the gut microbial community diversity or composition. In Ecuadorian children, single infections with *T. trichiura* were not associated with significant faecal microbiota differences compared to uninfected peers. By contrast, *Ascaris–Trichuris* co-infections were linked to reduced overall diversity, together with depletion of *Clostridium* sensu stricto and clostridial cluster IX taxa [63]. While the reduction in diversity is typically considered detrimental, the loss of these clostridial groups is often associated with heightened CRC risk, which may paradoxically reflect a protective shift. This underlines the dual and context-dependent nature of helminth–microbiome interactions, where the same infection can simultaneously generate potentially harmful and protective microbial outcomes.

Gobert and Atkinson [52] reported that *O*. *viverrini* infection in Thai participants led to only modest shifts in gut taxa (*Bacteroides* and *Clostridium* spp.) but significantly increased salivary α-diversity, with higher abundance of *Desulfovibrio* and *Dialister*. Both genera have been implicated in CRC development through their pro-inflammatory and genotoxic potential, underscoring the fact that parasite species and tissue niche (the gut versus oral cavity) can differentially influence cancer risk. Jenkins and Rathnayaka [62] found no significant changes in α-diversity among Sri Lankan participants with helminth infection, but β-diversity (a measure of compositional differences in microbial communities between samples) differed significantly, with enrichment of Enterobacteriaceae. This family has been consistently associated with intestinal inflammation and CRC progression, suggesting that even in the absence of diversity shifts, helminth infections may be associated with tumour-relevant microbial signatures. Similarly, in volunteers from non-endemic settings, *S. stercoralis* infection left α-diversity stable but shifted β-diversity and metabolomic pathways, including increased amino acid metabolites and reduced carbohydrate metabolism. Such changes have been associated with reduced SCFA production—metabolites critical for epithelial integrity and anti-carcinogenic protection that may contribute to a more permissive metabolic environment to CRC development [64,73].

Environmental and community-level factors also shaped outcomes. Tee and Er [65] reported that helminth-positive indigenous Malaysian individuals had Firmicutes-rich microbiomes, marked by overrepresentation of *Agathobacter rectalis* and *Blautia wexlerae*, but the effects varied significantly between villages, suggesting strong local modulation. In contrast, Nguélé and Mozzicafreddo [59] found that infected adults exhibited an increased Firmicutes/Bacteroidetes ratio, a pattern often associated with inflammatory bowel disorders (IBDs) including Crohn’s disease, which are conditions that may heighten CRC risk. By comparison, Taye and Mekonnen [44] observed enrichment of *Agathobacter*, a butyrate-producing genus within the Lachnospiraceae family, in Ethiopian children with *T*. *trichiura* infection. Given the anti-inflammatory and anti-carcinogenic roles of butyrate, this may represent a potentially protective microbial shift. Importantly, Martin and Djuardi [56] showed that albendazole treatment influenced microbiome composition in both infected and uninfected participants, with shifts in Actinobacteria and Bacteroidetes, highlighting the potential for drug effects to confound helminth–microbiome associations. Beyond treatment effects, dietary fibre intake and antibiotic history represent additional uncontrolled variables that were not systematically documented across the included studies. Fibre-rich diets independently enrich SCFA-producing Firmicutes and Ruminococcaceae, the very taxa most frequently attributed to helminth colonisation in this review, while antibiotic exposure can deplete commensal diversity in ways that may obscure or mimic helminth-associated dysbiosis. Geographic heterogeneity across studies spanning Southeast Asia, sub-Saharan Africa, South America, and non-endemic Western settings further introduces variation in baseline microbiome composition and co-infection burden that was not systematically accounted for in between-study comparisons. These observations illustrate that parasite species, infection burden, chronicity, host immunity, and environmental exposures collectively shape whether helminth-associated microbiome changes are protective or deleterious.

### 4.4. Synthesis and Implications for CRC

The relationship between helminths, the gut microbiome, and CRC cannot be reduced to a simple directional pathway. Instead, it operates through an integrated immune–microbiome–tumour axis in which helminth-driven immune polarisation, characterised by Th2/regulatory skewing, IL-10, TGF-β, and expanded Treg populations, simultaneously remodels microbial community structure, alters luminal metabolite production, and modulates epithelial barrier integrity. At the molecular level, epithelial barrier disruption may involve altered expression or localisation of tight junction proteins, including claudin-1, occludin and ZO-1, together with reduced MUC2, thereby increasing epithelial permeability, microbial translocation and CRC-relevant inflammatory signalling.

These interconnected shifts engage tumour-relevant processes, including genotoxin exposure, impaired immune surveillance, and chronic inflammatory signalling. Critically, the net outcome across this axis is not fixed: the same infection may enrich SCFA-producing commensals with anti-carcinogenic properties while concurrently permitting expansion of established CRC-associated taxa, depending on parasite species, infection intensity, chronicity, and host context. The evidence synthesised below maps this dualism across both protective and pro-tumourigenic trajectories.

On the one hand, they may enhance diversity, enrich SCFA-producing taxa, and promote immune homeostasis. For instance, *Parabacteroides* and Tenericutes expanded in patients with hookworm infection [53], *Agathobacter* increased in Ethiopian children [44], Firmicutes-rich profiles marked by *A*. *rectalis* and *B*. *wexlerae* were more common in helminth-positive Malaysian villages [65], and Paraprevotellaceae and Ruminococcaceae expanded in *S. stercoralis* infections [61,64]. These taxa are strongly associated with butyrate and propionate production: metabolites that reinforce epithelial integrity, suppress pro-inflammatory cytokines, and induce apoptosis in colonocytes, providing potential anti-carcinogenic benefits [70].

On the other hand, several studies showed enrichment of pathogenic and pro-inflammatory bacteria. *S. gallolyticus*, detected in Tanzanian women and Ethiopian children with *T. trichiura* infection, is a well-established CRC-associated bacterium [44,59]. Taken together, these two studies reveal the paradoxical role of *T*. *trichiura* in shaping microbial communities. On the one hand, Taye and Mekonnen [44] documented concurrent enrichment of *Agathobacter*, a butyrate-producing genus associated with epithelial protection and anti-inflammatory activity. On the other hand, Nguélé and Mozzicafreddo [59] highlighted the presence of *S*. *gallolyticus*, a species consistently linked to colorectal tumourigenesis, through its ability to adhere to colonic epithelial cells, promote chronic inflammation, and induce DNA damage that facilitates malignant transformation [78,82,83]. This contrast illustrates that *T. trichiura* infection has been associated with both protective shifts that support gut homeostasis and detrimental alterations potentially linked to CRC risk. The divergent outcomes most likely reflect differences in host immunity, infection intensity, and local environmental factors, reinforcing the need to view *Trichuris*–microbiome interactions as context-dependent rather than uniformly beneficial or harmful.

*Enterococcus*, *Flavonifractor*, and *Olsenella* were also enriched in individuals heavily infected in Liberia and Indonesia [60]. All three genera have been linked to CRC. *Enterococcus* is associated with DNA-damaging reactive oxygen species [84,85,86], while *Flavonifractor* has been connected to pro-inflammatory flavonoid metabolism. Emerging human evidence shows that the genus *Olsenella* may be enriched in CRC. For instance, a large prospective study of the oral microbiome reported that the presence of *Olsenella* was significantly associated with increased distal CRC risk (a hazard ratio of around 2.16; 95% CI 1.59–2.95) [87]. While mechanistic pathways remain under investigation, one proposed route involves bile-salt hydrolase activity in the family Atopobiaceae, which includes *Olsenella*. This activity may alter bile acid pools in favour of secondary bile acids that exert pro-tumourigenic effects, leading to epithelial stress and inflammatory signalling [88,89].

Beyond these taxa, other helminth-associated microbial shifts have also been linked to pathways relevant for colorectal carcinogenesis. *Ruminococcus torques*, a mucin-degrading species linked to barrier disruption, expanded in chronic *S. stercoralis* infections [61]. The loss of mucosal integrity associated with this expansion may contribute to microbial translocation and inflammation, which have been linked to tumour development [90,91]. At the molecular level, this barrier dysfunction may involve depletion or degradation of MUC2, a major structural component of the colonic mucus layer, together with impaired epithelial tight-junction integrity involving occludin, claudins and ZO-1 [92,93]. Disruption of these barrier components has been associated with increased paracellular permeability and exposure to microbial products, which may sustain CRC-relevant inflammatory signalling [93,94].

*A. lumbricoides* infections were associated with phylum-level shifts toward Firmicutes and Proteobacteria [58]. In severe cases, parasite metabolomes were enriched in amino acids, lipids, and nucleotide precursors [54], potentially creating nutrient-rich conditions favourable to tumourigenesis.

**Figure 2:**
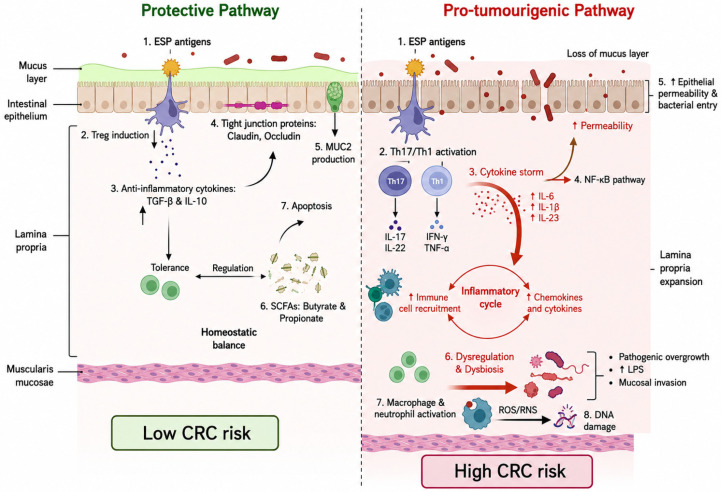
Dual effects of helminth infections on gut microbiome composition and colorectal cancer (CRC) development. The diagram depicts the contrasting protective (**left**) and tumour-promoting (**right**) mechanisms through which helminths influence host–microbiome interactions. The image was created in BioRender.com (accessed 27 October 2025).

In endemic, low-sanitation settings, many individuals harbour mild or moderate helminth infections, which are often associated with increased microbial diversity and enrichment of fermentative taxa that may stabilise the gut ecosystem [55,65]. In the same environments, however, subgroups with heavy or chronic infections exhibit microbial dysbiosis characterised by loss of beneficial commensals and enrichment of CRC-associated bacteria [59,60,61]. This spectrum helps reconcile why studies in endemic regions sometimes report protective effects, while others document pro-carcinogenic microbial shifts. The outcome is strongly dependent on infection intensity, chronicity, and host context. To provide a visual synthesis of these contrasting effects, Figure 2 depicts the proposed pathways through which helminth infections can exert either protective or pro-tumourigenic influences on the gut microbiome and CRC risk.

Protective pathway (left): (1) Helminth ESPs act on dendritic cells to promote (2) Treg induction. (3) Tregs release transforming growth factor-β (TGF-β) and IL-10, enhancing tolerance and regulation. (4) Tight junction proteins (claudin and occludin) and (5) MUC2 secretion maintain barrier integrity. (6) Commensals generate SCFAs, particularly butyrate and propionate, which fuel colonocytes and drive (7) apoptosis of abnormal cells. Together, these events sustain homeostasis and reduce CRC risk.

Pro-tumourigenic pathway (right): (1) ESPs stimulate dendritic cells, driving (2) Th17/Th1 activation. Th17 produces IL-17 and IL-22; Th1 secretes interferon-γ (IFN-γ) and tumour necrosis factor-α (TNF-α), leading to (3) a cytokine storm (↑ IL-6, IL-1β, and IL-23). (4) NF-κB activation disrupts (5) epithelial barrier integrity, increasing permeability and bacterial entry. (6) Dysbiosis follows, marked by loss of commensals and pathogenic overgrowth (*S*. *gallolyticus* and Enterobacteriaceae), increased lipopolysaccharide (LPS), and mucosal invasion. (7) Macrophage and neutrophil activation drive reactive oxygen and nitrogen species (ROS/RNS), causing (8) DNA damage. 

Chronic inflammation and genomic instability promote tumour initiation and progression, resulting in high CRC risk.

This dualism is not a contradiction but rather the central insight of this review, showing that helminth–microbiome interactions are not uniformly protective or harmful, but exist along a context-dependent continuum. For instance, *O. viverrini* infection in Thai participants led to modest shifts but increased salivary α-diversity [52], while albendazole treatment in Sri Lanka and Malaysia altered Actinobacteria and Bacteroidetes, even in uninfected participants [56,65], showing that treatment itself may act as a confounder. The immunosuppressive milieu generated by helminths, while protective in autoimmune settings, may simultaneously impair tumour immunosurveillance [57]. This aligns with high-impact human CRC metagenomic studies showing reproducible disease signatures such as *F*. *nucleatum*, Enterobacteriaceae, and *S*. *gallolyticus* across populations [40,77,78,95]. This overlap strengthens the argument that helminth-driven microbial shifts can converge mechanistically with established CRC-associated dysbiosis.

Beyond SCFAs, helminth-associated microbiome shifts may influence CRC-relevant biology through additional metabolite classes. Bile-salt hydrolase activity in *Olsenella* and related Atopobiaceae may skew luminal bile acid pools toward deoxycholic acid and lithocholic acid, which activate Wnt/β-catenin and NF-κB signalling and promote epithelial oxidative stress. Proteolytic fermentation products such as ammonia, phenols, and hydrogen sulphide can impair colonocyte respiration and induce genotoxic injury, while the nutrient-enriched parasite metabolome documented in severe *Ascaris* infections [54] may further favour pro-growth luminal conditions. Host genetic variation in Toll-like receptor 2 (TLR2), Toll-like receptor 4 (TLR4), and nucleotide-binding oligomerisation domain-containing protein 2 (NOD2) can modulate epithelial responses to these helminth-conditioned microbial signals, and chronic dysbiosis may leave epigenetic imprints, including promoter hypermethylation of tumour suppressor genes and butyrate-depletion-mediated loss of HDAC inhibition that compounds carcinogenic risk. Although none of the included studies assessed these mechanisms directly, they represent priority axes for future research and underscore that fully resolving the helminth–microbiome–CRC relationship will require longitudinal, multi-omics study designs that integrate parasitological, immunological, microbial, and host genomic data within the same cohort.

## 5. Limitations

A prominent limitation across most studies included in this systematic review is the small sample size, which reduces statistical power and the ability to detect subtle changes. For instance, Muslim and Aazmi [58] incorporated only four mature *Ascaris* worms and eight human faecal samples. Similarly, the recent Zanzibar cohort studied by Nguélé, Mozzicafreddo [59], only comprised a dozen women, with the author calling for larger follow-up cohorts, while Tran and Chaidee [61] compared 21 chronic *Strongyloides*-positive and 21 matched negative controls. Jenkins and Rathnayaka [62] also had a modest sample of 76 volunteers, limiting the generalisability of the observed changes in microbial beta diversity. The limited sample sizes underscore a significant deficiency in thoroughly evaluating helminth-induced gut microbiome diversity and CRC risk.

Another significant limitation is the restricted depth of microbiome analysis. Several studies relied on 16S rRNA sequencing or even older platforms (454 pyrosequencing). Nine of the seventeen studies used in this systematic review employed 16S rRNA gene sequencing [44,54,55,57,58,60,61]. These studies recognised the role of the 16S rRNA constraint in elucidating intricate microbial roles, advocating for metagenomic shotgun sequencing as an adjunctive method. Martin and Djuardi [56] encountered difficulties using obsolete sequencing systems, such as 454 pyrosequencing, which restricted the discovery of uncommon species in comparison to more advanced technologies like Illumina. Although Nguélé and Mozzicafreddo [59] used shotgun sequencing, a large number of reads, approximately 50%, remain unclassified due to incomplete reference databases. Similarly, Jenkins and Rathnayaka [62] and Jenkins and Pritchard [53] employed 16S-based approaches, which may not fully capture functional gene shifts or rare microbial taxa potentially linked to CRC development. These technical limitations highlight the necessity of utilising modern approaches to comprehensively assess microbiome makeup and function. Such approaches detect broad taxonomic changes but cannot assess microbial functional potential or rare taxa as comprehensively as shotgun metagenomics. Consequently, functional inferences drawn from taxonomic data, including SCFA production capacity, genotoxin output, and bile acid metabolism, represent plausible mechanistic hypotheses rather than confirmed biological activity, and should be interpreted with appropriate caution.

Study design issues also emerged, particularly regarding control of confounding variables and insufficient quality of methodologies; thus, there was minimal control for diet, antibiotic use, host immunity, or other environmental exposures that shape the gut microbiome. Klomkliew and Sawaswong [54] emphasised the deficiency of data on participants’ dietary habits, antibiotic consumption, and bacterial illness history, all of which may affect gut microbiota composition. Their inability to confirm findings using quantitative PCR, owing to sample limitations, constrained their conclusions. On the other hand, Muslim and Aazmi [58] only utilised microscopic analysis to detect parasite infections, lacking the sensitivity and specificity of molecular techniques. Lee and Tang [55] compared rural Malaysians with helminth infection to urban U.S. residents processed differently; differences in sample preservation and host demographics (unmatched ages and environments) could contribute to the observed microbial differences. Additionally, Lee and Tang [55] reported on the different storage protocols: rural Malaysian stools were stored in potassium dichromate at 4 °C for days, versus immediate freezing of U.S. samples. Lee and Tang [25] further illustrate the difficulty in disentangling dietary effects from helminth-associated changes in microbiota; despite identifying infection as the stronger determinant, the overlapping variables still complicate causal inferences. Jenkins and Pritchard [53] also faced challenges isolating infection effects from MS disease variability, given the limited samples and presence of comorbidities. Gobert and Atkinson [52] underscored how host-specific variables and microbial baseline differences across individuals and populations confound the direct attribution of gut changes to *Opisthorchis* infection alone. More broadly, the geographic spread of the included studies, spanning four continents with markedly different sanitation infrastructure, dietary traditions, co-infection profiles, study designs, helminth species, infection intensities, sequencing platforms and reported microbiome endpoints, introduces substantial between-study heterogeneity that limits direct cross-study comparisons, reduces the generalisability of pooled conclusions and precludes meta-analysis.

The complexity of studying interactions between multiple factors, such as co-infections or ecological variables, also posed challenges. Rosa and Supali [60] proposed augmenting sample sizes and integrating phylogenetic data into machine learning models to enhance prediction efficacy in multifactorial scenarios. Ducarmon and Hoogerwerf [51] in a controlled experimental study, found only temporary shifts in gut microbiota following *N*. *americanus* infection, suggesting that gut microbiome changes may be transient or masked by other dynamic gut processes.

Lastly, most of these studies were performed outside Africa, despite the increasing incidence of helminth infections in the region, mostly due to ongoing sanitation issues. However, while a recent Zanzibar study provides an African perspective, this is just one isolated context that cannot capture the continent’s epidemiological diversity.

## 6. Conclusions

This systematic review highlights the complex relationship between helminth infections and gut microbiome diversity, with significant implications for CRC risk. The findings reveal that helminths can profoundly reshape the composition and functional capacity of the gut microbiome, with outcomes ranging from enhanced stability and enrichment of SCFA-producing taxa to dysbiosis characterised by loss of protective commensals and expansion of pro-carcinogenic bacteria. This duality highlights that helminth–microbiome interactions exist along a spectrum, where mild or moderate infections may enhance microbial resilience, whereas heavy, chronic, or mixed infections foster inflammatory and oncogenic profiles.

Several studies reviewed, including those by [54,58,62], demonstrated shifts in dominant microbial taxa associated with helminth infection. These shifts included increases in families such as Verrucomicrobiaceae, Enterobacteriaceae, and Lachnospiraceae, enrichment of *S*. *gallolyticus* and *Ruminococcus torques*, and depletion of protective genera such as *Prevotella*, *Weissella*, and *Leuconostoc*. These microbial changes, particularly under high worm burdens or mixed infections, resemble those associated with early colorectal tumourigenesis, such as enriched DNA-damaging strains, depletion of protective SCFA producers, and increased microbial translocation across a compromised gut barrier [33,96,97,98]. The relationship between helminths and the gut microbiome is influenced by parasite species, infection intensity, co-infection status, and host immunity [54,55,60,62]. Context-dependent findings were also evident in studies such as those by Cooper and Walker [63] and Gobert and Atkinson [52], which reported minimal or site-specific microbial changes, highlighting that the effects of helminths cannot be generalised. While anthelmintic treatments can induce transient changes in the microbiome structure, evidence suggests that helminth-associated signatures may persist post-treatment. For instance, Lee, Tang [25] and Ducarmon and Hoogerwerf [51] showed that microbiome alterations linked to helminth exposure may re-establish after clearance, while [56,57] and Rosa and Supali [60] reported distinct microbial or immune configurations in individuals who remained infected. This effect suggests that helminth infections may leave long-term microbial and immunological imprints that sustain chronic low-grade inflammation, a known driver of CRC.

Additionally, helminths may indirectly modulate CRC risk through immune regulation. By potentially dampening tumour immunosurveillance or contributing to pro-inflammatory responses, helminths may be associated with microenvironments favourable to neoplastic transformation [53,57]. Given the reproducible overlap between helminth-associated taxa and CRC signatures identified in large-scale meta-analyses (e.g., *S*. *gallolyticus*, Enterobacteriaceae, and *F*. *nucleatum*) [40,77,78,95]. These findings strengthen the hypothesis that helminth-driven dysbiosis converges mechanistically with established pathways of colorectal carcinogenesis.

Future research should integrate functional metagenomics, metabolomics, and host–microbe immunological studies to unravel causal pathways linking helminth-induced microbial shifts to CRC. Longitudinal cohort studies in endemic populations will also be critical to determine whether infection history predicts long-term CRC outcomes. Importantly, this emerging body of evidence highlights the need for integrated public health approaches in helminth-endemic regions, where parasite control, nutritional interventions, and microbiome-targeted strategies could act synergistically to reduce the cancer burden.

## Figures and Tables

**Table 1:** Characteristics of included studies examining helminth–gut microbiome interactions in human populations (*n* = 17).

Study	Country (Study Setting)	Study Design	Population/ Study Group	Sample Size (*n*)	Sample Type	Helminth Species	Microbiome Profiling Method
[51]	Netherlands	Experimental	Healthy adults, experimental *Necator americanus* infection	20	Stool	*Necator americanus*	16S rRNA sequencing
[52]	Thailand	Cross-sectional	Adults with natural helminth infection	90	Stool, Saliva	Mixed helminths	16S rRNA sequencing
[53]	United Kingdom	Experimental	RMS ^1^ patients, experimental *Necator americanus* infection	71	Stool	*Necator americanus*	16S rRNA sequencing
[54]	Thailand	Cross-sectional	Children and young adults with natural *Ascaris lumbricoides* infection	49	Stool and helminth gut	*Ascaris lumbricoides*	16S rRNA sequencing
[55]	Malaysia	Cross-sectional	Adults with natural helminth infection	70	Stool	Mixed helminths	16S rRNA sequencing
[56]	Indonesia	Randomised controlled trial	Adults with natural helminth infection	150	Stool	Mixed helminths	16S rRNA sequencing
[57]	Indonesia	Randomised controlled trial	Adults with natural helminth infection	66	Stool	Mixed helminths	16S rRNA sequencing
[58]	Malaysia	Cross-sectional	Children with natural *Ascaris lumbricoides* infection	12	Stool and helminth gut	Mixed helminths	16S rRNA sequencing
[59]	Tanzania	Cross-sectional	Women of reproductive age with *Trichuris trichiura* infection	10	Stool	*Trichuris trichiura*	shotgun metagenomics
[60]	Liberia, Indonesia	Randomised controlled trial	Adults with natural helminth infection	402	Stool	Mixed helminths	16S rRNA sequencing and Shotgun metagenomics
[44]	Ethiopia	Cross-sectional	Children with natural helminth infection	138	Stool	Mixed helminths	16S rRNA sequencing
[61]	Thailand	Longitudinal	Adults with natural *Strongyloides stercoralis* infection	42	Stool	*Strongyloides stercoralis*	16S rRNA sequencing
[62]	Sri Lanka	Cross-sectional	Adults with natural helminth infection	76	Stool	Mixed helminths	16S rRNA sequencing
[25]	Malaysia	Longitudinal	Adults with natural helminth infection	67	Stool	Mixed helminths	16S rRNA sequencing
[63]	Ecuador	Cross-sectional	Children with natural *Trichuris trichiura* infection	97	Stool	Mixed helminths	16S rRNA sequencing
[64]	Italy	Longitudinal	Adults with natural *Strongyloides stercoralis* infection	31	Stool	*Strongyloides stercoralis*	16S rRNA sequencing
[65]	Malaysia	Longitudinal	Adults with natural helminth infection	650	Stool	Mixed helminths	Shotgun metagenomics

^1^ RMS, relapsing multiple sclerosis.

**Table 2:** Summary of gut microbiome alterations and related outcomes in human studies of helminth infection. Statistical measures reported reflect those used in the original studies and include *p*-values, odds ratios, and β coefficients; direct cross-study comparison is limited by methodological heterogeneity across the included studies.

Study	Study Aim (s)	Key Microbiome and Clinical Findings
[51]	To investigate temporal changes in the gut microbiota in response to different dosages (ranging from 50 to 150L3) of *Necator americanus* larvae in healthy young volunteers. To investigate temporal differences in the gut microbiota between healthy volunteers experiencing different numbers of clinical symptoms.	Alpha diversity: Increased bacterial richness during established infection (Chao1; *p* = 0.0174; weeks 8–20) Relative abundance: *Barnesiella* is more abundant in volunteers with higher GI ^2^ symptoms (*p* < 0.05) Microbiota stability/timepoints: Instability observed during early infection (weeks 0–8; *p* = 0.036) with recovery by week 20 (*p* = 0.004) Clinical outcomes: GI symptoms associated with microbial instability in early infection, with recovery by week 20
[52]	To determine if microbial changes are associated with parasite infection by profiling faecal and saliva microbiota of participants with helminth infection.	No significant changes in stool. Significant changes observed in saliva. Alpha diversity: Increase in saliva (Chao1; *p* = 0.042 at genus level; *p* = 0.026 at phylum level) Relative abundance: Increase: *Desulfovibrio* (*p* = 0.039), *Oxalobacter* (*p* = 0.022), Oxalobacteraceae (*p* = 0.022), *Dialister* (*p* = 0.027), and *Abiotrophia* (*p* = 0.031) Decrease: *Haemophilus* Specific to *Opisthorchis viverrini*: *Odoribacter* (*p* = 0.0066), *Phascolarctobacterium* (*p* = 0.031), and *Succinivibrio* (*p* = 0.05)
[53]	To investigate qualitative and quantitative changes in faecal bacterial composition in RMS ^3^ patients before and after experimental infection with *Necator americanus*, and to assess the therapeutic efficacy of live hookworm infective larvae in RMS patients.	Alpha diversity: Decrease in PBO ^4^ subjects at T9 compared to T pre (*p* < 0.05) Beta diversity: Higher in N+ (*Necator americanus* infected) subjects at T9 compared to PBO (*p* = 0.048) Relative abundance: *Parabacteroides* significantly expanded in N+ individuals with no relapses Clinical outcomes: 51% of N+ subjects showed no new CNS ^5^ lesions vs. 28% of PBO subjects
[54]	To investigate the gut bacteriomes of *Ascaris lumbricoides* helminths and stool samples of patients with different infection intensities, as well as to characterise the metabolomes of *Ascaris lumbricoides* in heavy and light ascariasis cases.	Alpha diversity: Chao1 richness is higher in heavily infected patients (*p* < 0.05) Beta diversity: Bray–Curtis distance did not distinguish between infected and uninfected individuals (PERMANOVA ^6^ *p* < 0.001) Relative abundance: *Prevotella is* more abundant in ascariasis patients (*p* < 0.05); *Streptococcus* is more abundant in heavily infected patients; *Lactococcus* is more abundant in lightly infected patients Clinical outcomes: Increased levels of essential biomolecules in heavily infected patients
[55]	To compare the gut microbiota of individuals with helminth infection and individuals who were not colonised by helminths, and to develop new approaches to examine the interactions of microbes with each other and their hosts.	Alpha diversity: Increase in helminth-colonised individuals (*p* = 0.04) Beta diversity: ANOSIM ^7^ (R = 0.18; *p* = 0.04) Relative abundance: Increase in Paraprevotellaceae, Mollicutes, Bacteroidales, and Alphaproteobacteria in helminth-colonised individuals; increase in *Bifidobacterium* in helminth-negative individuals
[56]	To characterise the joint effects of several predictors, including helminth infection and treatment, on each bacterial category.	Relative abundance: Actinobacteria increased (OR ^8^ = 1.57; 95% CI ^9^: 1.05–2.35) and Bacteroidetes decreased (OR = 0.35; 95% CI: 0.18–0.70) in infected subjects receiving albendazole vs. PBO Alpha diversity: No significant differences in Shannon diversity or richness between infected and uninfected groups (pre- and post-treatment) Beta diversity: Bray–Curtis dissimilarity remained stable (61%) between pre- and post-treatment samples Clinical outcomes: STH ^10^ prevalence reduced in the albendazole group (21.7% vs. 54.3%; *p* < 0.001) at 21 months
[57]	To characterise the association between gut microbiome composition and immune responses, and to examine the effect of helminth infections on this relationship.	Alpha diversity: Higher diversity associated with increased IFN-γ ^11^ response (β = 0.95; 95% CI: 0.15–1.75; *p* = 0.056) Relative abundance/effect sizes: Bacteroidetes negatively associated with IL-10 ^12^ response (β = −1.96; 95% CI: −3.05 to −0.87; *p* = 0.002); Actinobacteria associated with decreased TNF-α ^13^ (β = −1.55; 95% CI: −2.87 to −0.22; *p* = 0.024); Firmicutes associated with IL-5 ^14^ response post-treatment (β = −1.52; *p* = 0.024) Clinical outcomes: Deworming did not significantly alter microbiome–cytokine associations
[58]	To characterise the gut microbiota of the SHT parasite *Ascaris lumbricoides* and compare it to the gut microbiota of their human hosts.	Alpha diversity: Lower in *Ascaris* (*p* = 0.006) Beta diversity: Significant separation between *Ascaris* and human gut microbiota (Bray–Curtis *p* = 0.002; Jaccard *p* = 0.004) Relative abundance: Firmicutes higher in *Ascaris* (84.2%; *p* = 0.03); *Clostridium* significantly higher in *Ascaris* (*p* = 0.001) Timepoints: Samples obtained 3 days post-albendazole treatment
[59]	To investigate the effects of *Trichuris trichiura* infection on the gut microbiome composition and function in women of reproductive age from Pemba, Tanzania.	Relative abundance: *Prevotella* is higher in healthy participants; *Weissella cibaria*, *Leuconostoc citreum*, and *Leuconostoc lactis* are lower in infected individuals; Proteobacteria are higher in infected samples; *Treponema succinifaciens* and *Streptococcus gallolyticus* are higher in infected samples; overall fungal abundance is higher in infected participants Functional analysis: Cholesterol metabolism and pathogenic infection pathways are higher in infected samples (*p* < 0.05) Firmicutes/Bacteroidetes ratio: Higher in infected participants
[60]	To enhance understanding of human gut microbiome interactions with STHs in Liberia and Indonesia, despite varying gut microbiome structures among individuals.	Alpha diversity: Higher richness and evenness (89.5 vs. 82.1, *p* = 5.8 × 10^−10^; 2.8 vs. 2.4, *p* = 0.0008) Beta diversity: Higher in infected individuals (0.52 vs. 0.43, *p* < 10^−5^); lower with self-clearing over time (2010: 0.54 vs. 0.60, *p* = 0.008) Relative abundance: *Prevotella* dominant (32%) Functional analysis: Arachidonic acid metabolism and K00560 were enriched; 11 Kos ^15^ were depleted Clinical outcomes: Self-clearing individuals were older (51.1 vs. 21.4 years, *p* = 0.012)
[44]	To identify and explain differences in microbial communities between STH-infected and non-infected Ethiopian school children.	Alpha diversity: Lower in *Trichuris trichiura*-infected children (*p* < 0.05) Beta diversity: Differences by infection status (PERMANOVA *p* < 0.01) Relative abundance/correlation: *Agathobacter was* higher in infected children (adjusted *p* = 0.001) and positively correlated with egg counts (*p* < 0.05)
[61]	To explore the impact of chronic *Strongyloides stercoralis* infection on the gut microbiome and microbial activity in a longitudinal study.	Alpha diversity: No significant difference Beta diversity: Difference in unweighted UniFrac (R^2^ = 0.037; *p* = 0.028) Relative abundance: *Ruminococcus torques* group is over-represented in chronic infection Timepoints: T0 (baseline), T1 (1 year), and T2 (4 months post-treatment) Clinical outcomes: Reduction in *Ruminococcus torques* group after treatment; increased carbohydrate metabolism
[62]	To explore the qualitative and quantitative differences in microbial community profiles between individuals with patent infections by parasitic nematodes, uninfected individuals, and those who have received regular anthelmintic treatment.	Alpha diversity: No significant difference (Shannon; *p* = 0.65) Beta diversity: Higher in individuals with (H+) and treated for helminth (Ht) (*p* = 0.04) Relative abundance: Verrucomicrobiaceae and Enterobacteriaceae are higher in H+; Leuconostocaceae are higher in helminth-negative (H−); Bacteroidaceae are higher in Ht; and *Akkermansia muciniphila* is higher in H+ individuals
[25]	To identify the important factors influencing the gut microbiota and to determine the relative impact of helminth infection on host responses and the microbiota.	Microbiome: Helminth infection influenced gut microbiota more than diet; OTUs ^16^ associated with fibre intake and *Trichuris trichiura* burden Clinical/immune: Zinc was higher in Orang Asli (*p* = 1.67 × 10^−9^); iron was higher in urban (*p* = 0.002); CD8+ was higher and naïve CD4+ was lower; and natural killer cells were reduced post-treatment Gene expression: Deworming altered 654 genes (FDR ^17^ < 10%) and reduced the *Trichuris trichiura* burden associated with increased IL20RB ^18^
[63]	To investigate the effects of *Trichuris trichiura* infections on faecal microbiota and to determine if anthelmintic treatment reverses these changes towards a microbiota composition similar to that of uninfected individuals.	Alpha diversity: Lower in mixed infections (Shannon; *p* = 0.004) Relative abundance: *Clostridium sensu stricto* was higher in uninfected individuals (4.1% vs. 1.5%; *p* = 0.013); uncharacterised clostridial cluster IX bacteria were higher in uninfected individuals (2.3% vs. 0.6%; *p* = 0.026) Clinical outcomes: 100% cure of STH infections at 7 and 21 days post-treatment
[64]	To determine the effects of chronic, monospecific infections by *Strongyloides stercoralis* on the faecal microbiome and metabolome of human volunteers from a non-endemic area and to establish whether these effects are reversed by anthelmintic treatment.	Alpha diversity: Higher in *Strongyloides stercoralis* positive (S+) subjects (Simpson’s index: F = 5; *p* = 0.03; evenness: F = 4.2; *p* = 0.05) Beta diversity: Lower in S+ subjects (R = 0.11; *p* = 0.04) Relative abundance: Leuconostocaceae, Ruminococcaceae, Paraprevotellaceae, and *Peptococcus are* higher in S+; *Bacteroides* is lower in S+ Metabolites: Alanine, formate, lysine, and leucine are higher in S+ (F ≈ 4.2–4.5; *p* = 0.03–0.05)
[65]	To investigate the interactions between helminth infections and the human gut microbiome in indigenous Malaysians using shotgun metagenomics.	Alpha diversity: Higher richness in infected subjects (*p* = 2.5 × 10^−5^); no significant change post-treatment Beta diversity: Small effect size between pre- and post-treatment (ADONIS ^19^: R^2^ = 0.014; *p* = 0.001; ANOSIM: R = 0.072; *p* = 0.001) Relative abundance: Specific replicating taxa associated with helminth infection Timepoints: Longitudinal changes assessed post-albendazole treatment

^2^ GI, gastrointestinal; ^3^ RMS, relapsing multiple sclerosis; ^4^ PBO, placebo; ^5^ CNS, central nervous system; ^6^ PERMANOVA, permutational multivariate analysis of variance; ^7^ ANOSIM, analysis of similarity; ^8^ OR, odds ratio; ^9^ CI, confidence interval; ^10^ STH, soil-transmitted helminth; ^11^ IFN-γ, interferon-gamma; ^12^ IL-10, interleukin-10; ^13^ TNF-α, tumour necrosis factor-alpha; ^14^ IL-5, interleukin 5; ^15^ KOs, KEGG orthology terms; ^16^ OTUs, operational taxonomic units; ^17^ FDR, false discovery rate; ^18^ IL20RB, interleukin-20 receptor subunit beta; ^19^ ADONIS, permutational multivariate analysis of variance based on distance matrices.

## Data Availability

All data analysed in this systematic review are derived from publicly available published studies. No new datasets were generated, and no additional files accompany this manuscript.

## Abbreviations

The following abbreviations are used in this manuscript:

16S rRNA	16S ribosomal ribonucleic acid
ADONIS	Permutational multivariate analysis of variance based on distance matrices
Akt	Protein kinase B
ANOSIM	Analysis of similarity
AUC	Area under the curve
BFT	Bacteroides fragilis toxin
CD4^+^	Cluster of differentiation 4-positive
CD8^+^	Cluster of differentiation 8-positive
CI	Confidence interval
CNS	Central nervous system
CRC	Colorectal cancer
DNA	Deoxyribonucleic acid
ESPs	Excretory-secretory products
ETBF	Enterotoxigenic Bacteroides fragilis
FDR	False discovery rate
GI	Gastrointestinal
GRADE	Grading of Recommendations, Assessment, Development and Evaluations
H−	Helminth-negative individuals
H+	Individuals infected helminth
HDAC	Histone deacetylase
HR	Hazard ratio
Ht	Individuals treated for helminth individuals
IARC	International Agency for Research on Cancer
IBD	Inflammatory bowel disease
I-FABP	Intestinal fatty acid-binding protein
IFN-γ	Interferon-gamma
IL-10	Interleukin-10
IL-17	Interleukin-17
IL-1β	Interleukin-1 beta
IL20RB	Interleukin-20 receptor subunit beta
IL-22	Interleukin-22
IL-23	Interleukin-23
IL-5	Interleukin-5
IL-6	Interleukin-6
KEGG	Kyoto Encyclopaedia of Genes and Genomes
KOs	KEGG orthology terms
LMICs	Low- and middle-income countries
LPS	Lipopolysaccharide
M2-like macrophages	Alternatively activated macrophage-like phenotype
MS	Multiple sclerosis
MUC2	Mucin 2
N+	*Necator americanus*-infected individuals
NF-κB	Nuclear factor kappa-light-chain-enhancer of activated B cells
NK	Natural killer
NOD2	Nucleotide-binding oligomerisation domain-containing protein 2
NTDs	Neglected tropical diseases
OR	Odds ratio
OTUs	Operational taxonomic units
PBO	Placebo
PCR	Polymerase chain reaction
PERMANOVA	Permutational multivariate analysis of variance
PHA	Phytohaemagglutinin
PI3K	Phosphoinositide 3-kinase
pks	Polyketide synthase
PRISMA	Preferred Reporting Items for Systematic Reviews and Meta-Analyses
qPCR	Quantitative polymerase chain reaction
RCT	Randomised controlled trial
RMS	Relapsing multiple sclerosis
RNS	Reactive nitrogen species
ROC	Receiver operating characteristic
ROS	Reactive oxygen species
S+	Individuals with *Strongyloides stercoralis*
SCFAs	Short-chain fatty acids
STHs	Soil-transmitted helminths
TEER	Transepithelial electrical resistance
TGF-β	Transforming growth factor-beta
Th1	T-helper type 1
Th17	T-helper type 17
Th2	T-helper type 2
TLR2	Toll-like receptor 2
TLR4	Toll-like receptor 4
TNF-α	Tumour necrosis factor-alpha
Treg	Regulatory T cell
UniFrac	Unique fraction metric
ZO-1	Zonula occludens-1
α-diversity	Alpha diversity; a measure of species richness and evenness within individual samples
β-diversity	Beta diversity; a measure of compositional differences in microbial communities between samples
